# Supplementary material for: The differential immune responses to COVID-19 in peripheral and lung revealed by single-cell RNA sequencing
Source: Cell Discov. 2020 Oct 20;6:73. doi: 10.1038/s41421-020-00225-2 (PMC7574992; doi:10.1038/s41421-020-00225-2)
Supplement: Supplementary file 1 — Supplementary Figures [file 41421_2020_225_MOESM1_ESM.pdf]

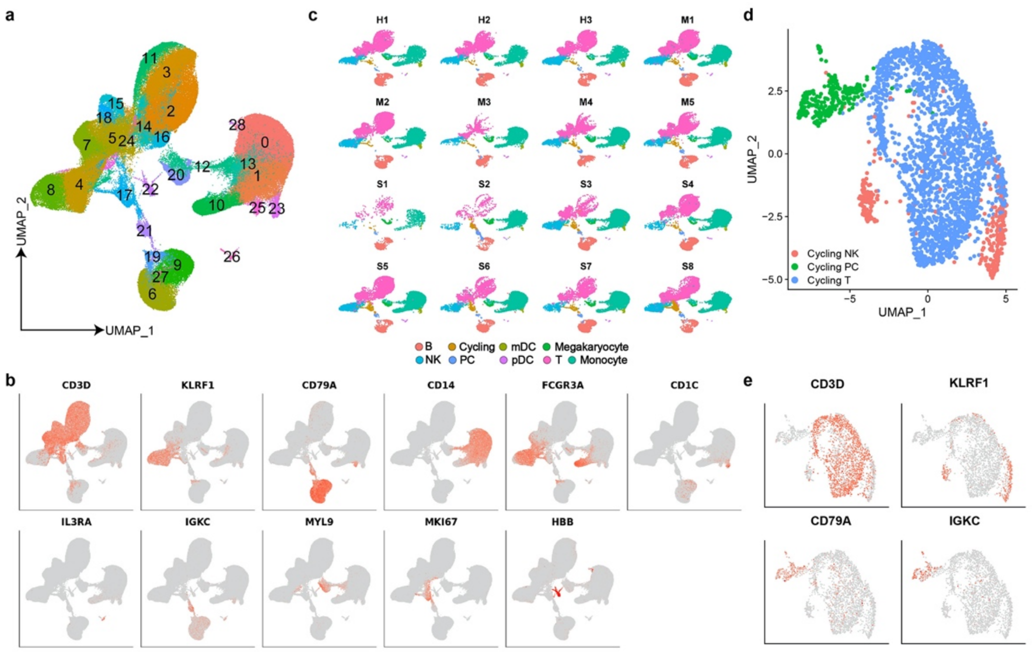

Supplementary Fig. S1 The clustering and annotation data related to Figure 1

(a) 29 cell clusters are identified in PBMCs and visualized in UMAP.

(b) Immune cell markers defining the major cell lineages are projected in UMAP from (a).

(c) Cells in each studied subject are projected in the UMAP, including 3 controls (HC1 to HC3), 5 patients in mild group (M1 to M5), and 8 patients in severe group (S1 to S8).

(d) Cycling cells in PBMCs are re-clustered and contain three major cell types.

(e) The specific markers defining each cycling cell types are projected in UMAP from (d).

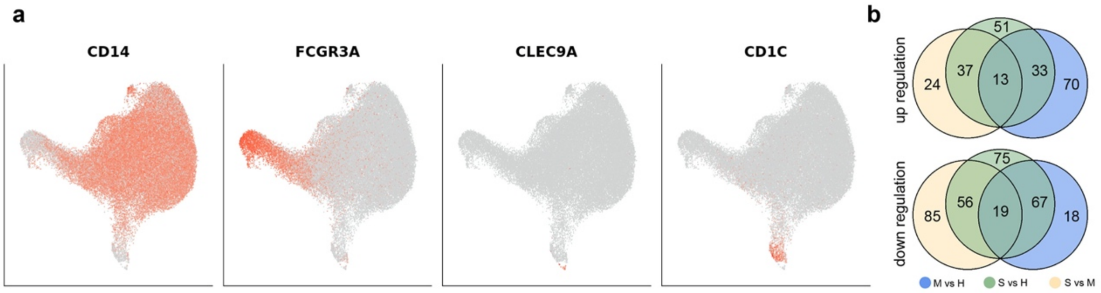

Supplementary Fig. S2 The blood myeloid cell types annotation and DEG analysis related to Figure 2

(a) Cell markers defining the major myeloid cell types in PBMCs are projected in UMAP plots.

(b) Venn diagram shows up-regulated and down-regulated DEGs in comparisons of blood $CD14^+$ monocyte between mild cases and controls (M vs H), severe cases and controls (S vs H), severe and mild cases (S vs M). (Fold change > 1.5 or < -1.5, adjust $P < 0.01$.

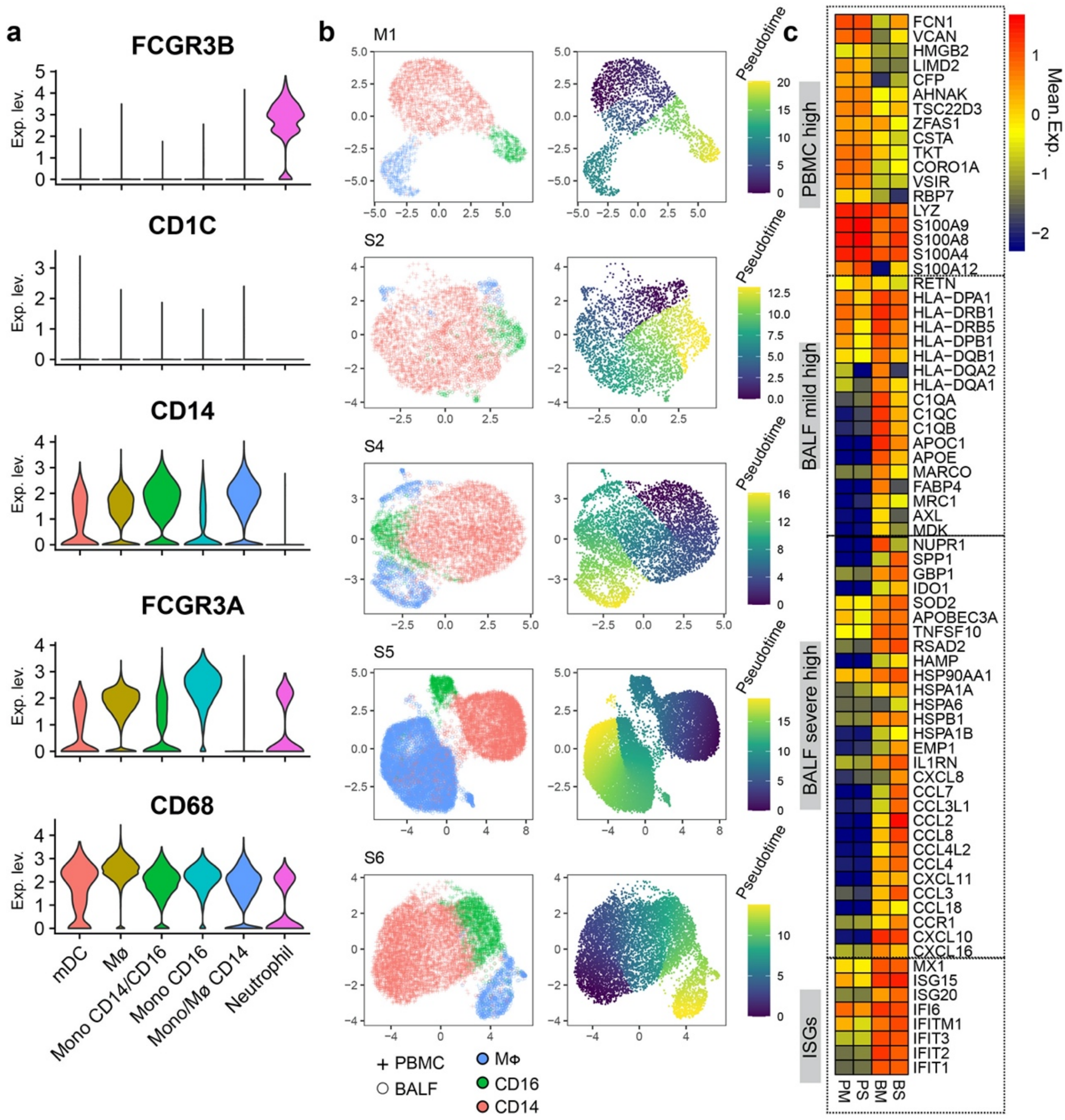

Supplementary Fig. S3 Integrated analysis of myeloid cells in PBMC and BALF (related to Figure 3)

(a) Violin plots show the expression of canonical myeloid cell markers in different cell types.

(b) Differentiation trajectory of the blood monocytes and BALF monocyte-macrophages from other five COVID-19 patients not shown in Figure 3, analyzed independently.

(c) Heatmap shows the selected DEGs in monocyte-macrophage comparisons as indicated (Fold change > 1.5 or < -1.5, adjust $P < 0.01$) (PM: Peripheral cells of mild cases; PS: Peripheral cells of severe cases; BM: BALF of mild cases; BS: BALF of severe cases).

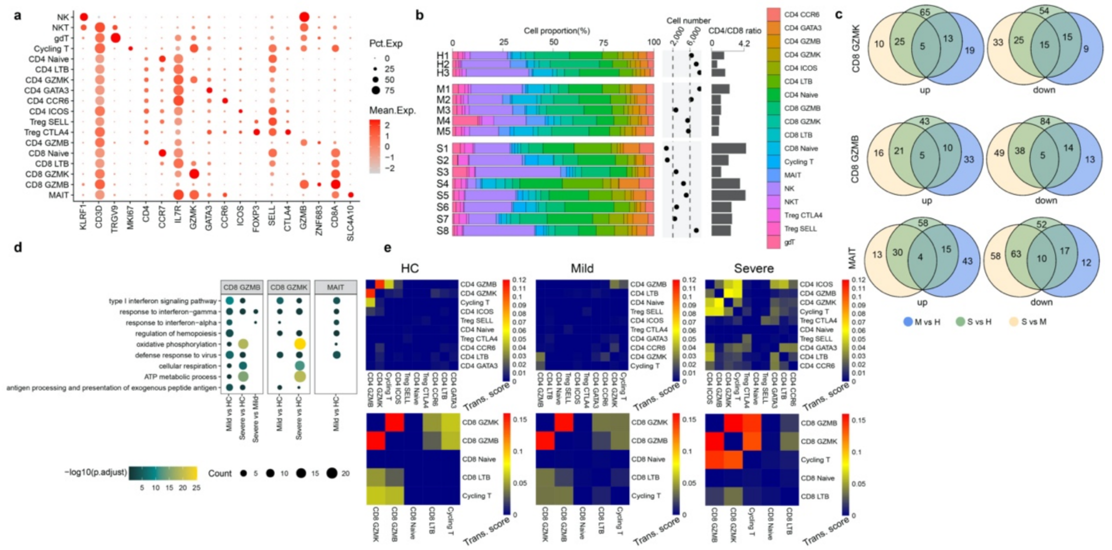

Supplementary Fig. S4 Analysis of peripheral NK and T cells (related to Figure 4)

(a) Expression of cell markers defining various NK and T cell subsets in Figure 4 are displayed.

(b) The bar plot shows the proportions of different T and NK clusters in PBMCs from individual subjects. The cell numbers and ratios of CD4 / CD8 T-cells are listed to the right-side.

(c) Venn diagram shows up-regulated and down-regulated DEGs in peripheral CD8 GZMK, CD8 GZMB and MAIT cells comparisons as indicated (H - controls, M - mild COVID-19, S - severe COVID-19).

(d) Enrichment of GO biological process (BP) terms for DEGs presented in (c).

(e) Heatmap shows the transition score quantified by pairwise STARTRAC-tran indices between T cell clusters in two patient groups and controls, respectively.

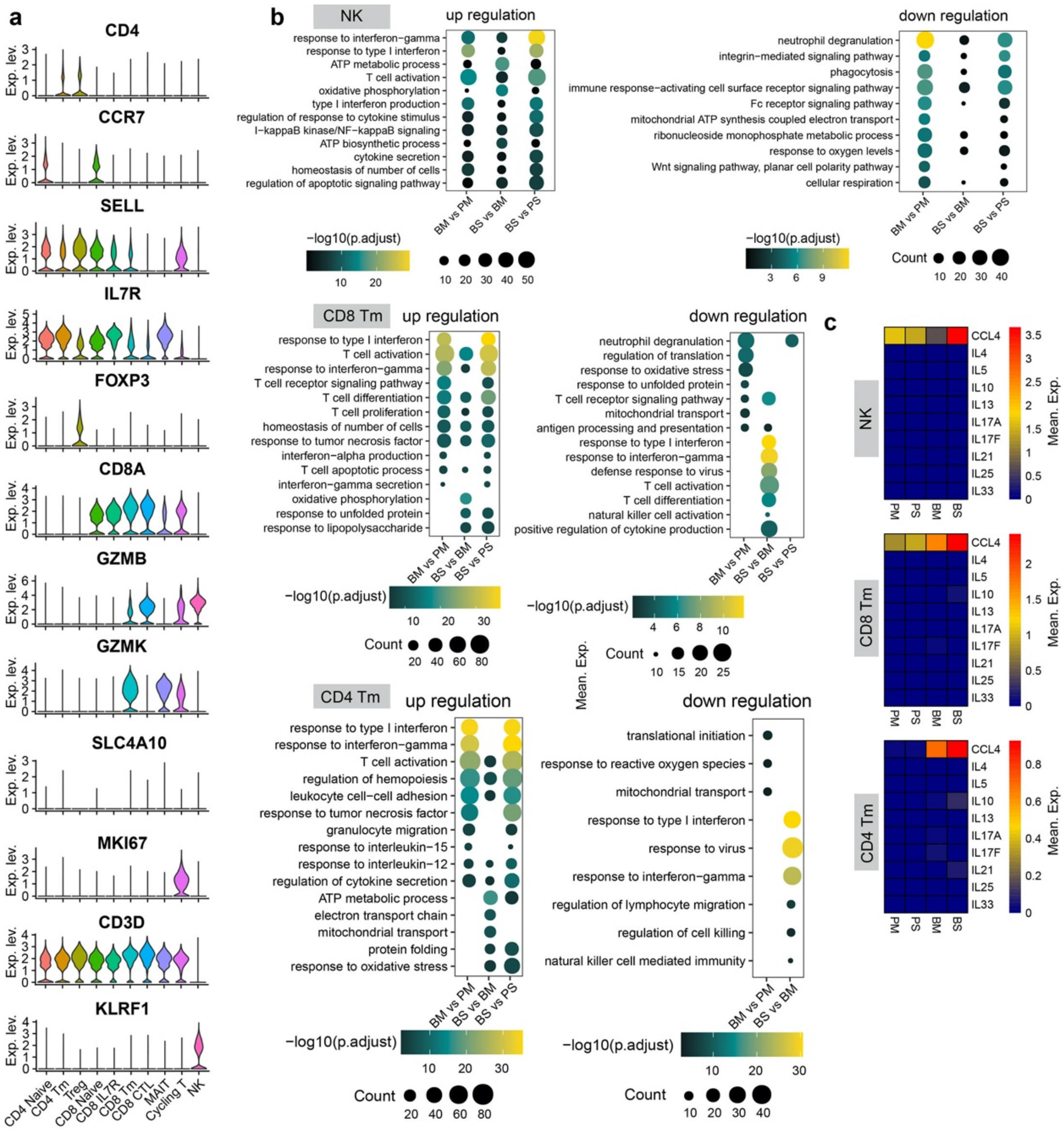

Supplementary Fig. S5 Integrated analysis of T cell compartments in PBMC and BALF (related to Figure 5)

(a) Expression of cell markers defining various NK and T cell subsets in Figure 5 are displayed.

(b) Enrichment of GO biological process (BP) terms for up-regulated and down-regulated DEGs in NK, CD4 Tm and CD8 Tm comparisons as indicated (Selected terms are shown, adjusted *P* < 0.01) (BM - BALF in mild cases; BS - BALF in severe cases; PM - PBMC in mild cases; PS - PBMC in severe cases).

(c) Heatmap shows the expression levels of selected T-cell relevant cytokines in various NK and T cell clusters from PBMC and BALF.
